# Impact of isolation method on doubling time and the quality of chondrocyte and osteoblast differentiated from murine dental pulp stem cells

**DOI:** 10.7717/peerj.3180

**Published:** 2017-06-14

**Authors:** Rohaya Megat Abdul Wahab, Nur Akmal Mohamed Rozali, Sahidan Senafi, Intan Zarina Zainol Abidin, Zaidah Zainal Ariffin, Shahrul Hisham Zainal Ariffin

**Affiliations:** 1 Department of Orthodontic, Universiti Kebangsaan Malaysia, Kuala Lumpur, Malaysia; 2 School of Biosciences and Biotechnology, Universiti Kebangsaan Malaysia, Bangi, Selangor, Malaysia; 3 Centre for Graduate Studies, Research Resources Centre, Cyberjaya University College of Medical Sciences, Cyberjaya, Selangor, Malaysia; 4 School of Biology, Universiti Teknologi MARA, Shah Alam, Selangor, Malaysia

**Keywords:** Dental pulp, Enzymatic digestion, Outgrowth, Osteoblast, Stem cell, Chondrocyte

## Abstract

**Background:**

Stem cells are normally isolated from dental pulps using the enzymatic digestion or the outgrowth method. However, the effects of the isolation method on the quality of the isolated stem cells are not studied in detail in murine models. The aim of this study was to compare the matrices secreted by osteoblast and chondrocytes differentiated from dental pulp stem cells isolated through different means.

**Method:**

DPSC from murine incisors were isolated through either the outgrowth (DPSC-OG) or the enzymatic digestion (DPSC-ED) method. Cells at passage 4 were used in this study. The cells were characterized through morphology and expression of cell surface markers. The cells’ doubling time when cultured using different seeding densities was calculated and analyzed using one-way ANOVA and Tukey’s multiple comparison post-test. The ability of cells to differentiate to chondrocyte and osteoblast was evaluated through staining and analysis on the matrices secreted.

**Results:**

Gene expression analysis showed that DPSC-OG and DPSC-ED expressed dental pulp mesenchymal stem cell markers, but not hematopoietic stem cell markers. The least number of cells that could have been used to culture DPSC-OG and DPSC-ED with the shortest doubling time was 5 × 10^2^ cells/cm^2^ (11.49 ± 2.16 h) and 1 × 10^2^ cells/cm^2^ (10.55 h ± 0.50), respectively. Chondrocytes differentiated from DPSC-ED produced  2 times more proteoglycan and at a faster rate than DPSC-OG. FTIR revealed that DPSC-ED differentiated into osteoblast also secreted matrix, which more resembled a calvaria.

**Discussion:**

Isolation approaches might have influenced the cell populations obtained. This, in turn, resulted in cells with different proliferation and differentiation capability. While both DPSC-OG and DPSC-ED expressed mesenchymal stem cell markers, the percentage of cells carrying each marker might have differed between the two methods. Regardless, enzymatic digestion clearly yielded cells with better characteristics than outgrowth.

## Background

Many methods have been applied in the isolation of dental pulp stem cells (DPSC) such as outgrowth, pre-treatment with enzyme followed by outgrowth, or pre-treatment with an enzyme prior to digestion with another enzyme. Different combinations of enzymes have also been used in DPSC isolation, which include dispase/collagenase (Ellis et al., 2014) and trypsin/collagenase (Balic et al., 2010). Most of the time, collagenase is included in the enzyme mixture as dental pulp consists predominantly of collagen (Yildirim, 2013a). However, regardless of how the isolation takes place, the basic principle of stem cell isolation from tissues has always involved either the migration of cells out of tissue explants or enzyme digestion of the tissues to obtain single cell suspensions.

DPSC isolated through outgrowth and enzymatic digestion yielded different types of cell populations due to the nature of the isolation process (Karamzadeh, Eslaminejad & Aflatoonian, 2012). Both types of isolation involved cell selection through the ability of cells to continue proliferating *in vitro* through the act of passaging. However, the outgrowth method yielded DPSC by manipulating the ability of cells to migrate out of tissues, which could occur through contact inhibition of cell locomotion (Reig, Pulgar & Concha, 2014; Scarpa et al., 2013). This mechanism of cell migration depended on the cell density and space where the cells move from the explant, which was high in cell number, towards the empty spaces around the explant. Meanwhile, isolating DPSC through enzymatic digestion was a more straightforward process. Early passages of DPSC isolated through enzymatic digestion would be contaminated with mature cells as all cells were released once the pulp matrices were digested (Ellis et al., 2014; Guimarães et al., 2011). Population of DPSC could be obtained as mature cells mostly stay at the G_0_ phase of cell cycle (Cooper, 2000). The higher proliferative ability of DPSC allowed the cells to dominate the culture after several passages.

DPSC is a type of mesenchymal stem cell which can be differentiated to form bone. Bone is comprised of hard structure, which provides support, and soft structure known as cartilage that prevents bone friction and provides cushion. Chondrocytes and osteoblasts are cells from mesenchymal origin that are involved in the maintenance of cartilage and hard bone, respectively. These two types of cells originate from the same progenitor, which is the osteochondral progenitor cell. The expression of transcription of factors SOX9 or RUNX2 determine the progenitor cell fate as to whether to further differentiate into chondrocyte or osteoblast (Phimphilai et al., 2006; Yang et al., 2011).

Osteoblasts are involved in the bone remodelling process by working side by side with osteoclasts in order to maintain bone homeostasis. Osteoblasts control matrix mineralization by secreting vesicles containing calcium and phosphate, which are components making up the bone. Chondrocytes meanwhile, secrete proteoglycan matrix, which make up cartilages. Chondrocytes are also involved in bone elongation through a process known as endochondral ossification, which takes place at the region of bone called the epiphyseal growth plate (Goldring, 2012).

Studies involving tissue specific stem cells require steps to obtain cells from the tissue. A lot of methods have been used and this variation prevents direct comparison of data for the cells’ potency. This study aimed to compare the ability of DPSC isolated through outgrowth and enzymatic digestion to proliferate *in vitro* and to analyze the quality of chondrocyte and osteoblast differentiated from the DPSC.

## Materials and Methods

### Cell culture

Dental pulp was isolated from 6–8 weeks ICR strain murine (*n* = 3) incisor with approval from the institution’s animal ethical committee (FST/2015/SHAHRUL/25-MAR.664-MAR.-2015-FEB.-2018-AR-CAT2). The mandibles were dissected and placed in ice cold PBS. Under stereomicroscope, the incisor was separated from the surrounding alveolar bone and the dental pulp was gently removed from interior of the incisor. The pulp was isolated through either enzymatic digestion or outgrowth method. Briefly, for the enzymatic digestion approach, the pulp was digested with 0.8 mg/mL collagenase 1A for an hour with gentle shaking. For the outgrowth method, the pulp tissue was minced into small pieces, placed in 6 well plate and the cells were allowed to migrate out of the tissues. These cells were cultured until passage 4 in DMEM supplemented with 20% (v/v) fetal bovine serum and 1% (v/v) penicillin-streptomycin, which will be here after referred to as proliferation medium. Each experiment was conducted using 3 biological replicates (3 different mice) with 3 technical replicates.

### Cell differentiation

Passage 4 cells were used for differentiation analysis to chondrocyte and osteoblast. Differentiation was carried out for 21 days with medium change of every 3 days. Chondrocyte differentiation was conducted using the micromass approach (Yamashita, Krawetz & Rancourt, 2008). Briefly, 10 µL of 1 × 10^7^ cell/mL dental pulp stem cells were placed in the middle of a 24-well plate and left to adhere for 2 h. Chondrocyte differentiation medium (Zenbio, USA) was slowly added as to not disturb the aggregate. For osteoblast differentiation, cells were seeded at a density of 2 × 10^4^ cells/cm^2^ in 96-well plate and cultured in proliferation medium supplemented with 50 µg/mL ascorbic acid and 10 mM *β*-glycerophosphate.

### Reverse transcriptase polymerase chain reaction

Total RNA was isolated and converted to cDNA using a Grandscript cDNA Synthesis Kit (PCR Biosystems, UK). RT-PCR was conducted by means of touchdown PCR using a Techne® Prime thermal cycler using the primers and steps previously published in detail (Nur Akmal et al., 2014).

### Cell proliferation and doubling time calculation

The cell proliferation assay was conducted by means of MTT. At a specified day, cells were cultured in proliferation medium containing 0.5 mg/mL MTT and incubated at 37 °C in humidified atmosphere with 5% CO_2_ for 4 h. The formazan crystals that were formed were dissolved in DMSO:glycine buffer (pH 10.5) at a ratio of 8 to 1. Absorbance was measured using Microplate Reader Model 680 (Biorad, USA) at 570 nm with 655 nm as the reference filter.

Doubling time was calculated based on the exponential phase of a growth curve. Cells were seeded at density of 1 × 10^2^ cells/cm^2^, 5 × 10^2^ cells/cm^2^, 1 × 10^3^ cells/cm^2^, 5 × 10^3^ cells/cm^2^ and 1 × 10^4^ cells/cm^2^. At a fixed day, cells were detached and trypan blue cell exclusion assay was used to calculate the number of cells. Growth curve was plotted to determine the exponential phase of the cells at each seeding density. The doubling time was calculated using an online doubling time calculator (http://www.doubling-time.com/compute.php).

### Toluidine blue and von Kossa staining

Toluidine blue and von Kossa were used to stain cells differentiated to chondrocyte and osteoblast, respectively. Prior to staining, cells were fixed using 10% (v/v) ice cold formalin in PBS for 30 min as done previously by Choi et al. (2008). Toludine blue staining was conducted by exposing the fixed cells to 0.1% (v/v) toluidine blue for 15 min. The cell morphology was observed under a microscope.

For von Kossa staining, cells were rinsed using deionized water and incubated in 5% (w/v) AgNO_3_ for 30 min. The brown coloured calcium nodules were developed by 5 min of incubation in 5% (w/v) NaCO_3_ in 25% (v/v) formalin and the excess, unbound AgNO_3_ was removed by washing with 5% (w/v) Na_2_O_3_S_2_ for 2 min. Densitometric analysis of the stained nodules was conducted using ImageJ 1.46r software.

### Sulphated glycosaminoglycan assay

Sulphated glycosaminoglycan assay was conducted using dimethylmethylene blue assay (Biocolor, UK). Cell aggregates were harvested and kept in −80 °C until they were ready to be analyzed. The assay was conducted according to manufacturer’s protocol with slight modification. Briefly, cell aggregates were digested in papain enzyme solution for 3 h at 65 °C with shaking. The digested aggregates were centrifuged at 10,000 g for 10 min and incubated in 1 mL of dye reagent for 30 min. The glycosaminoglycan-dye complex was pelleted through centrifugation at 12,000 rpm for 10 min. The resulting pellet was left to dissolve in 500 µL dissociation reagent for another 10 min. Approximately 200 µL of the sample was pipetted into 96-well plate and absorbance reading was taken at 655 nm. The absorbance value was compared to the absorbance of known concentrations of chondroitin-4-sulphate from bovine trachea.

### Fourier transform infrared (FTIR) spectroscopy

Cells were harvested in ammoniated water (50 mM ammonium bicarbonate, pH 8), lyophilized and analyzed as KBr pellets using Fourier Transformed Infrared (Luppen et al., 2003). Briefly, 100 mg samples were ground to homogeneity in 1 mg KBr powder using an agate mortar and pestle and pressed into thin pellets. Scans were taken at the rate of 64 cm^−1^ at 2.0 cm^−1^ interval using the Perkin Elmer Spectrum BX. Baseline correction, spectrum smoothing, and normalization were done using a Spectrum v5.0.1 (Perkin Elmer Instruments LLC). Overlapping peaks were resolved using second derivative methodology and the curve fitted with a mixed Gaussian and Lorentzian function using PeakFit v4.12. The degree of matrix mineralization was calculated using integrative area of *v1, v3* phosphate (1,200–900 cm^−1^) and amide I (1,720–1,590 cm^−1^), and the carbonate:phosphate ratio was calculated based on the region of *v2* carbonate (890–850 cm^−1^) (Kato et al., 2001). The degree of crystal maturity was calculated as the peak area of 1,030:1,110 (Farlay et al., 2010). Calvaria used as a positive control for well-mineralized bone was obtained from the same mice from which the pulp was isolated.

### Statistical analysis

Statistical analysis was performed using paired *T*-test or one-way ANOVA with post-hoc Tukey test. *p* < 0.05 was considered as significant.

## Results

### Characterization of dental pulp stem cells isolated through outgrowth and enzymatic digestion method

**Figure 1:**
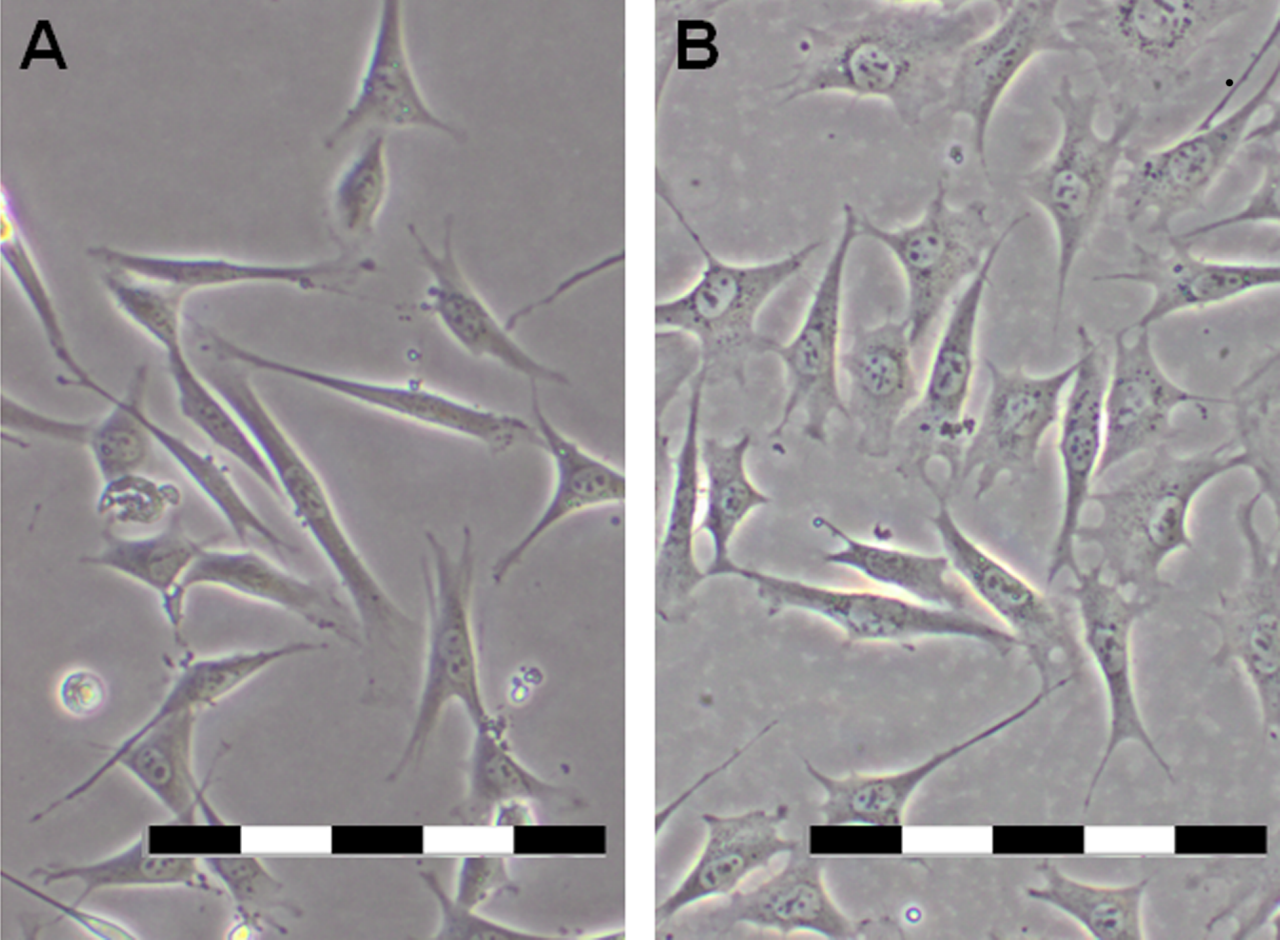
Morphology of (A) DPSC-OG and (B) DPSC-ED at passage 4. Both types of cells exhibited fibroblast-and stellate-like morphology. Scale bar: 200 µm.

**Figure 2:**
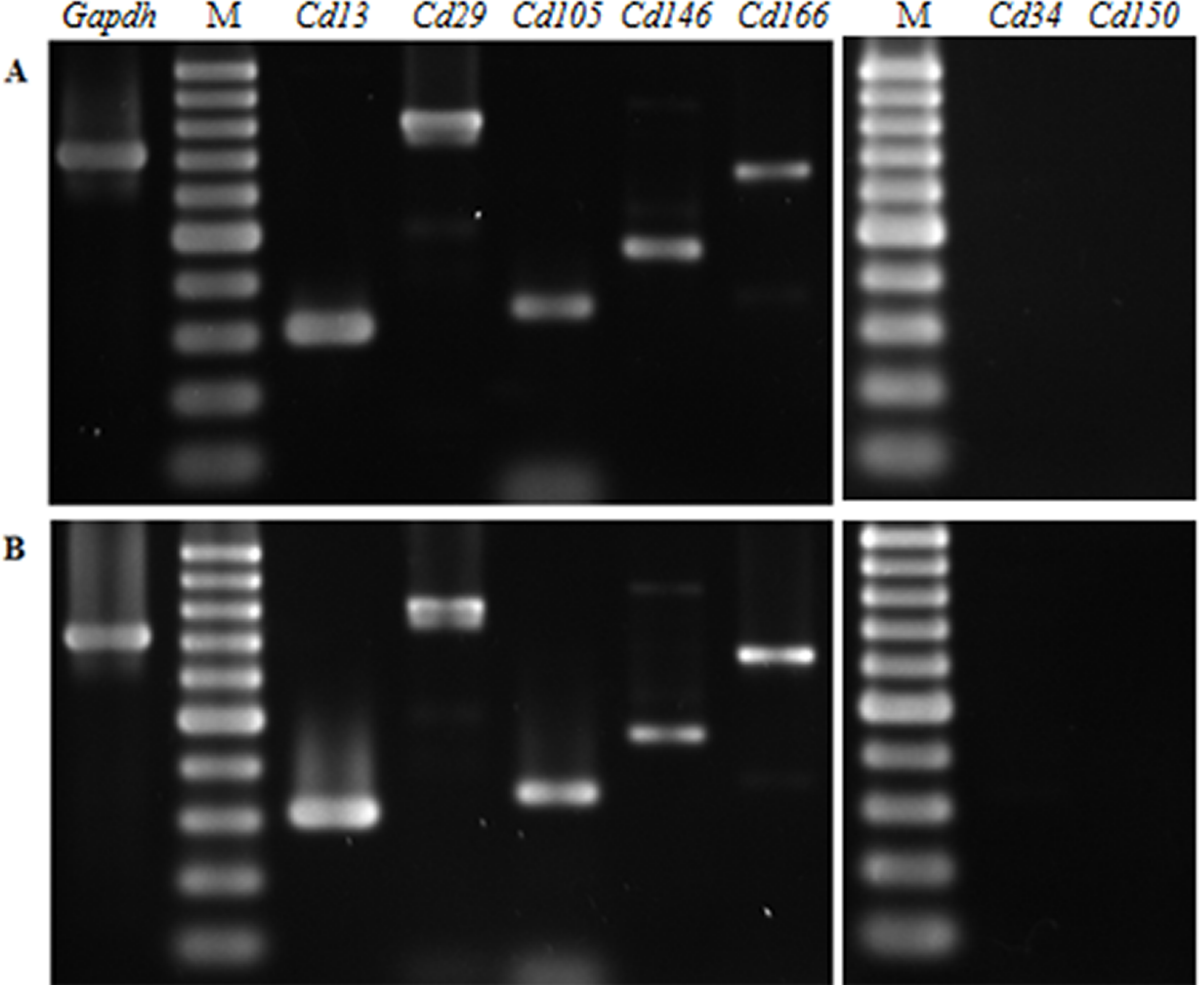
Gene expression profiles of passage 4 DPSC-OG (A) and DPSC-ED (B) using reverse transcriptase PCR. Agarose gel electrophoresis profile of dental pulp stem cells isolated through enzymatic digestion (DPSC-ED) and outgrowth (DPSC-OG) using dental pulp stem cell markers (*Cd13*, *Cd29*, *Cd105*, *Cd146*, *Cd166*), and hematopoietic stem cell markers (*Cd38*, *Cd150*). Housekeeping gene *Gadph* was used as an internal control for the RT-PCR reaction. Cells isolated through both methods expressed mesenchymal dental pulp stem cell markers and no expression of hematopoietic stem cell markers was detected. M: 100 bp ladder.

Dental pulp stem cells (DPSC) at passage 4 were used in this study. The cells showed the same fibroblast- and stellate-like morphology at passage 4 regardless of the method of extraction used (Fig. 1). Reverse transcriptase PCR (RT-PCR) revealed that the passage 4 DPSC isolated through outgrowth (DPSC-OG) and enzyme digestion (DPSC-ED) expressed DPSC marker *Cd13*, *Cd29*, *Cd105*, *Cd146*, *Cd166,* but not hematopoietic stem cell markers as shown in Fig. 2.

**Figure 3:**
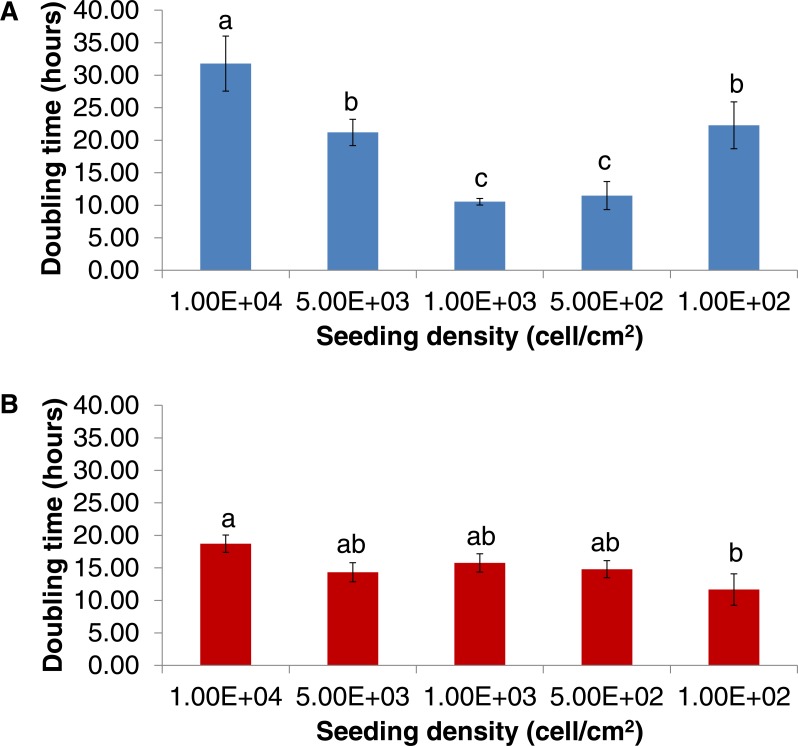
Doubling time of DPSC-OG (A) and DPSC-ED (B). Cells were seeded at different seeding densities. Doubling time was calculated based on the exponential phase of a growth curve. Comparisons between different densities were made using one-way ANOVA followed by Tukey post-hoc test. Values plotted were mean doubling time ± standard deviation. *p* < 0.05 was considered as significant as denoted by different letters.

Other than differentiation potency, one of the sought after characteristics was their ability to be expanded using a low starting number within a short period. Doubling time analyses revealed that the doubling time increased as the seeding density increased (Fig. 3). The shortest doubling times recorded for DPSC-OG cells were 10.55 ± 0.50 h (when seeded at 1 × 10^3^ cell/cm^2^) and 11.49 ± 2.16 h (when seeded at 5 × 10^2^ cell/cm^2^) with no significant difference between the two densities (*p* > 0.05). DPSC-ED can be seeded at lower density compared to DPSC-OG, which was at 1 × 10^2^ cell/cm^2^ with a doubling time of 10.55 h ± 0.50.

### Differentiation of dental pulp stem cells to chondrocyte

**Figure 4:**
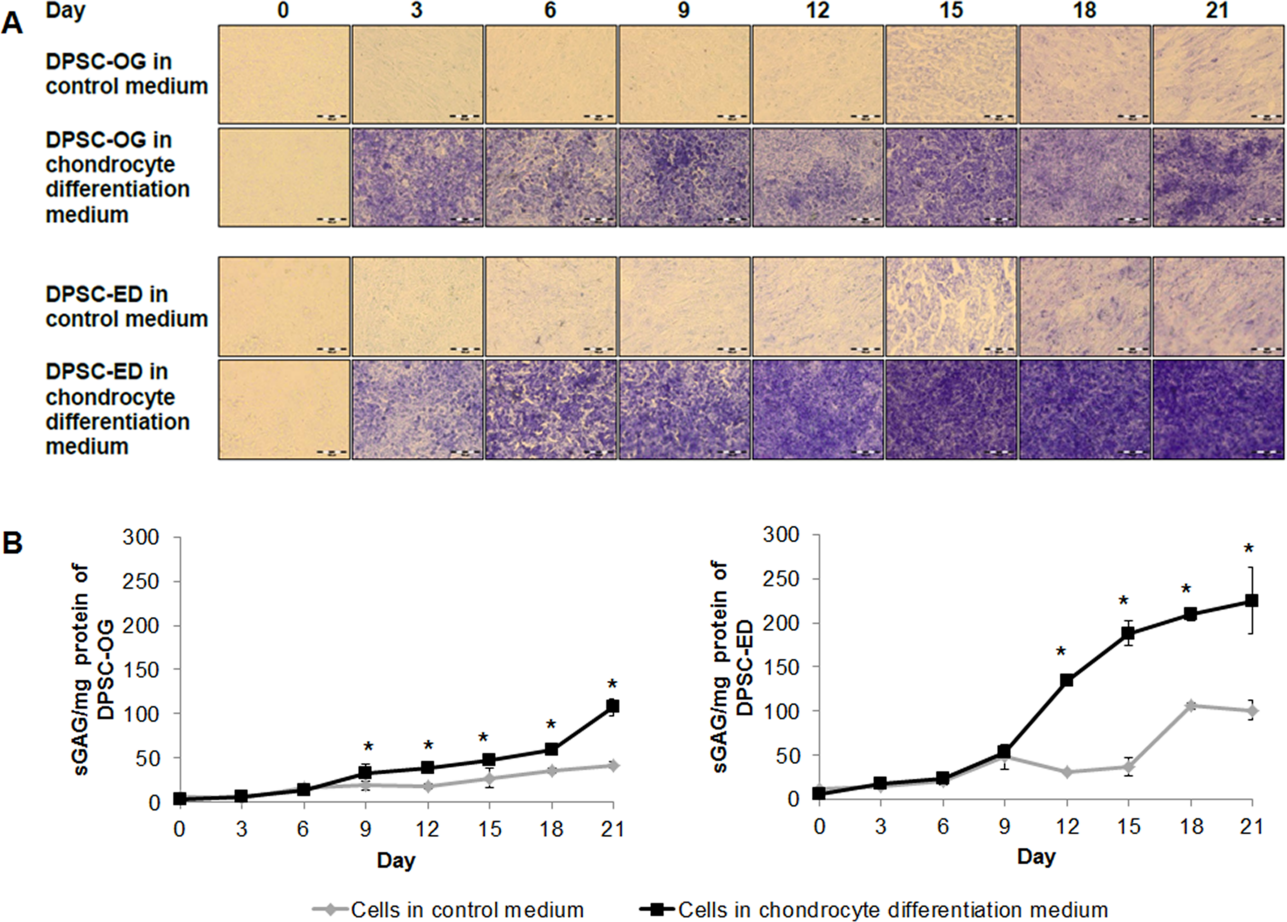
Toluidine blue staining (A) and sGAG content (B) of DPSC-OG and DPSC-ED during chondrocyte differentiation. DPSC-OG and DPSC-ED was cultured as micromass. Differentiation was carried out using cells in complete proliferation medium as the negative control. (A) The sections of culture stained purple showed the presence of proteoglycan. Scale: 100 µm. (B) sGAG contents was plotted as mean sGAG ± standard deviation. *p* < 0.05 was considered as significant (*).

Chondrocyte differentiation was conducted by means of micromass. The presence of proteoglycan matrix secreted by chondrocyte was detected by toluidine blue. Toluidine blue is a metachromatic dye, which will stain sulphated glycosaminoglycan (sGAG), one of the many components that made up proteoglycans, purple. Figure 4A shows differentiated and undifferentiated (control) DPSC-OG and DPSC-ED that had been stained with toluidine blue. The control cells were also stained light purple because toluidine blue can bind to the negatively charged phosphate on DNA (Sridharan & Shankar, 2012), and this was treated as background noise. The staining revealed that the intensity of the purple colour was becoming higher in differentiated DPSC-ED as compared to DPSC-OG starting around day 12. However, as toluidine blue is a type of qualitative staining, the content of proteoglycan was quantified using sGAG assay. sGAG assay showed that the sGAG content starts to increase significantly at day 9 for DPSC-OG and day 12 for DPSC-ED (Fig. 4B). The result also showed that the amount of sGAG secreted by DPSC-ED were approximately 2 times higher than DPSC-OG. This result was consistent with toluidine blue staining. These findings suggested that there might either be a higher percentage of DPSC-ED differentiating to chondrocytes or the chondrocytes were upregulated in function and thus, were able to secrete more proteoglycans.

### Differentiation of dental pulp stem cells to osteoblast

**Figure 5:**
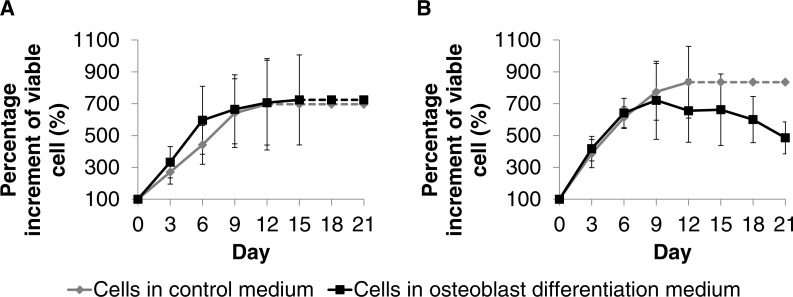
Proliferation analyses for DPSC-OG (A) and DPSC-ED (B) during differentiation to osteoblast. DPSC-OG and DPSC-ED was cultured in 96 well plate at 2 × 10^4^ cell/cm^2^ in proliferation medium (control medium) and proliferation medium supplemented with 50 µg ascorbic acid and 10 mM *β*-glycerophosphate (osteoblastic differentiation medium). The MTT assay was conducted every 3 days for a period of 21 days. The dotted line signifies undetermined percentage increment in viable cells due to unreadable MTT absorbance as the microplate upper limit had been reached. The highest absorbance value read by the microplate reader was ∼3.2 for both samples. Percentage of viable cells increment was fixed at 100% at Day 0. Comparison between the percentage increment of viable cells in the control medium and osteoblastic differentiation medium was conducted using paired *T*-test. * denotes significant difference (*p* < 0.05). Values were plotted as mean percentage of viable cell increment ± standard deviation.

**Figure 6:**
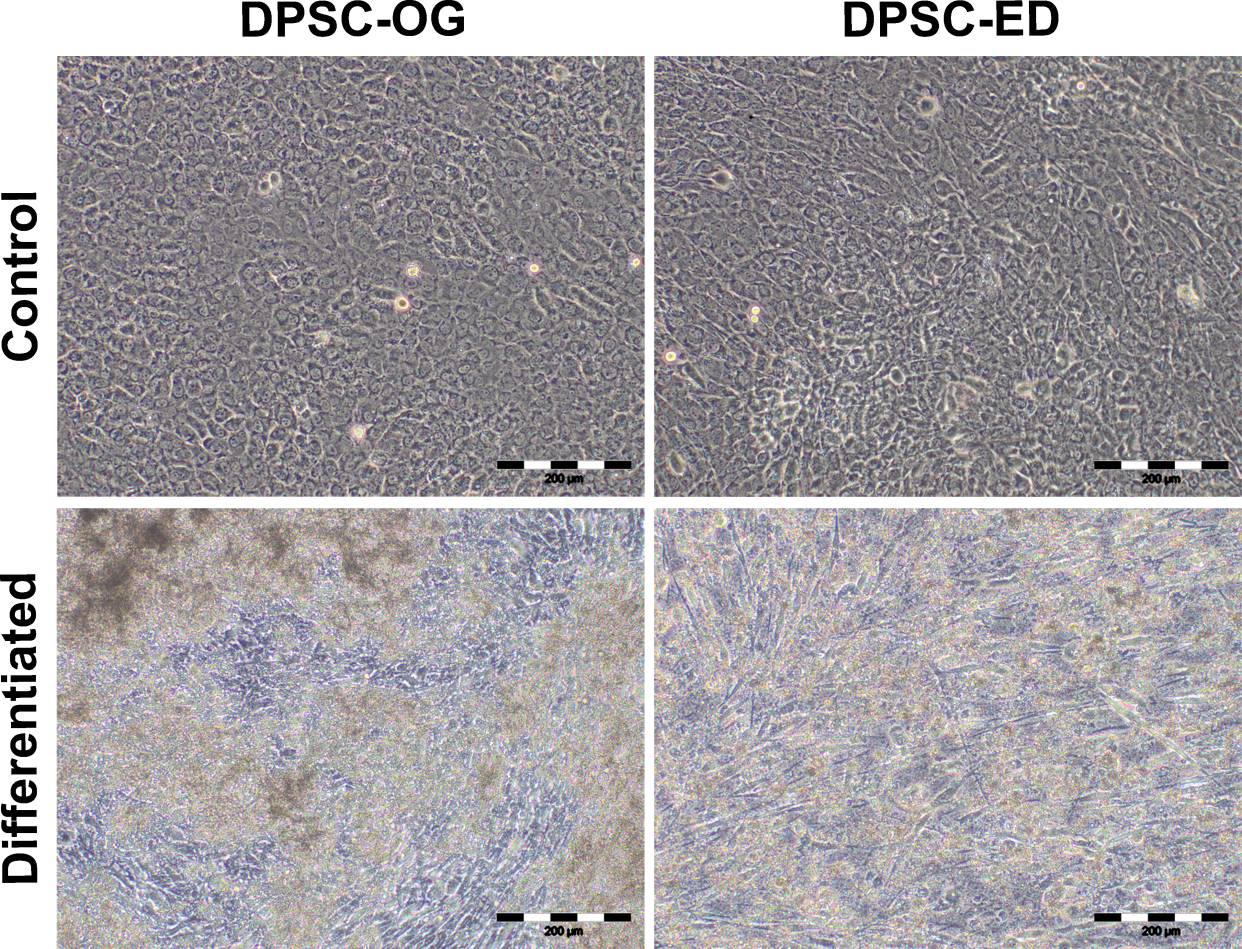
Morphology of DPSC-OG and DPSC-ED at day 21 of osteoblastic differentiation. DPSC was cultured in proliferation medium (control) and osteoblastic differentiation medium (differentiated). After 21 days, there was a decrease in the confluency of DPSC-ED in differentiation medium as compared to control. No visible change in the confluency was detected between DPSC-OG in the control and differentiation medium. Scale: 200 µm.

DPSC-OG and DPSC-ED was differentiated into osteoblasts as monolayer culture on a tissue culture treated plate. Proliferation analysis using MTT assay revealed that DPSC-OG and DPSC-ED were still able to proliferate when cultured in differentiation medium with no significant difference (*p* < 0.05) with the cells cultured in control medium (Fig. 5). However, cell proliferation for DPSC-ED in differentiation medium started to decrease by day 12. As there was a possibility for the decrease in proliferation to be due to more bone matrix deposition that makes MTT reagent less accessible to cell cytoplasm, microscopic images of cells were taken at Day 21 of differentiation (Fig. 6). Based on the images, there was a clear distinction between the cell confluency of differentiated DPSC-ED as compared to control. This finding was congruent with the proliferation assay result using MTT, which showed there was decrement in the number of cells for differentiated DPSC-ED as compared to the undifferentiated control.

**Figure 7:**
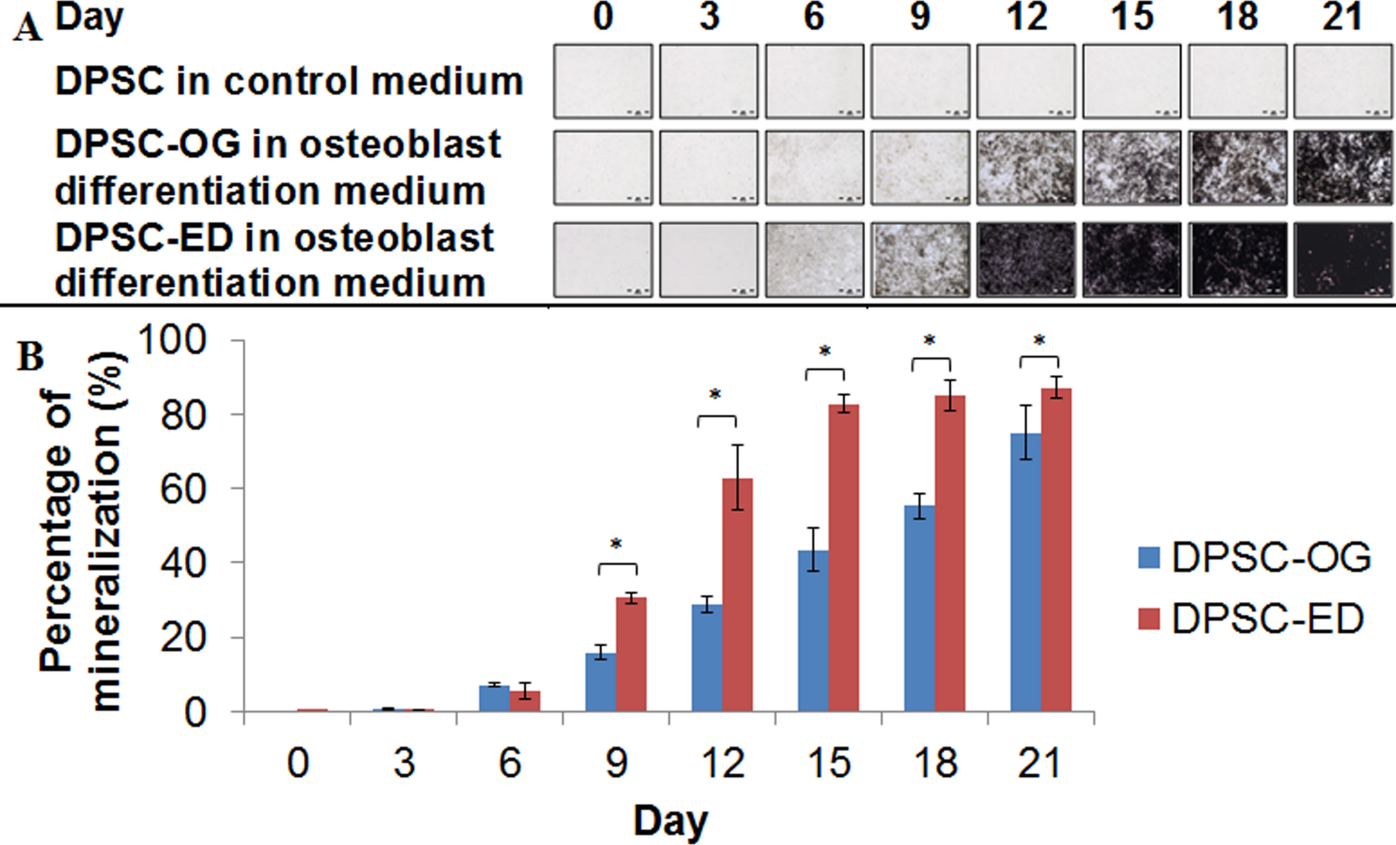
Von Kossa staining (A) and quantification of the mineralization area (B). Matrices secreted by dental pulp stem cells isolated through outgrowth (DPSC-OG) and enzymatic digestion (DPSC-ED) during osteoblastic differentiation were stained using von Kossa. The area of mineralization as depicted by the brown colour was measured and normalized to the control cell. Mineralization percentage was compared between DPSC-OG and DPSC-ED through paired *T*-test and *p* < 0.05 was considered significant (*). Values were represented by percentage of mineralization ± standard deviation.

The time-course staining of the differentiated DPSC showed increase in the brown colour from the von Kossa staining of calcified nodules for both DPSC-OG and DPSC-ED (Fig. 7). Von Kossa staining showed that DPSC-ED formed mineralized matrix faster than DPSC-OG. These results were in agreement with the proliferation assay, which showed that osteoblast differentiated from DPSC-OG may have become embedded in the matrix later than DPSC-ED, resulting in faster growth arrest of DPSC-ED than DPSC-OG (Fig. 5). The same findings were obtained for human DPSC isolated from permanent teeth, which showed that human DPSC-ED formed higher amount of mineralized tissue than DPSC-OG (Karamzadeh, Eslaminejad & Aflatoonian, 2012).

**Figure 8:**
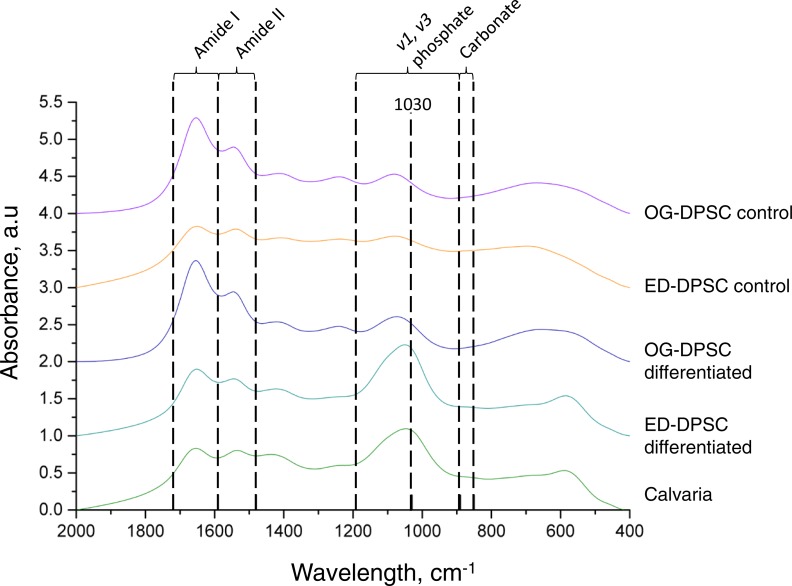
FTIR spectrum of dental pulp stem cell during differentiation process to osteoblast. Cells were cultured in control media and osteoblast differentiation medium. After 21 days, cells were harvested, freeze-dried, and analyzed as KBr pellets. The absorbance profile of DPSC-ED cultured in osteoblast differentiation medium was most similar to that of calvaria. An increase in the peak at 1,030 cm^−1^ could be seen when compared to cells in the control medium, signifying accumulation of highly crystalline, matured apatite during osteoblastic differentiation.

FTIR spectra of DPSC-OG and DPSC-ED were compared with the spectrum of a calvaria as a control for well-crystallized apatites (Fig. 8). Compared to differentiated DPSC-OG, differentiated DPSC-ED showed the most similar spectrum when compared to calvaria. The appearance of peak at 1,030 cm^−1^, which showed the existence of stoichiometric apatite (Farlay et al., 2010), can be seen clearly in differentiated DPSC-OG and DSPC-ED compared to undifferentiated cells. Amide I and II in the spectra showed the presence of collagen. The amide I band is the most intense absorption in collagen and is directly related to the backbone conformation of collagen (Figueiredo, Gamelas & Martins, 2012).

The degree of bone mineralization of differentiated DPSC-OG and DPSC-ED was calculated by normalizing the *v1, v3* phosphate: organic matrix amide I integrative area ratio of differentiated cells to undifferentiated cells. It was found that the degree of bone mineralization was significantly higher in differentiated DPSC-ED as compared to calvaria and DPSC-OG. DPSC-OG, meanwhile, showed no significant differences when compared to the mineralization degree in calvaria (Table 1). This result agreed with von Kossa staining, which showed higher staining intensity in DPSC-ED than DPSC-OG (Fig. 7). Carbonate peak was not detected in both DPSC-OG and DPSC-ED cultured in control medium or in differentiated DPSC-OG (Fig. 8). Differentiated DPSC-ED showed no significant difference in the level of carbonate substitution as calvaria as indicated by the carbonate:phosphate ratio (Table 1). The maturity of the apatite crystals, which was the measurement of the index of transformation of poorly crystallized apatite to well-crystallized stoichiometric apatite (Farlay et al., 2010), was comparable to calvaria for both differentiated DPSC-OG and DPSC-ED (Table 1).

**Table 1:** FTIR data analysis.

Sample	Degree of bone mineralization^1^	Relative amount of carbonate in bone mineral^2^	Apatite maturity index^3^
Calvaria	3.696541 ± 1.122246^a^	0.007106 ± 0.005761396	1.502549 ± 0.239476
DPSC-OG (control medium)	0	–	0
DPSC-ED (control medium)	0	–	0
DPSC-OG (osteoblast differentiation medium)	3.148611 ± 1.316780^a^	–	1.279091 ± 0.065186
DPSC-ED (osteoblast differentiation medium)	7.049769 ± 1.539965^b^	0.000509 ± 0.000040	1.124369 ± 0.094341

**Notes.**

1 Area of *v1, v3* phosphate (1,200–900 cm^−1^): area of organic matrix amide I (1,720–1,590 cm^−1^).

2 Ratio of area of *v2* carbonate peak (890–850 cm^−1^): area of phosphate peak.

3 Second derivative spectra was used to measure the ratio of peak area 1,030:1,110.

a, b One way ANOVA was conducted to compare between calvaria and dental pulp stem cells isolated through outgrowth (DPSC-OG) and enzymatic digestion (DPSC-ED) in terms of degree of bone mineralization and apatite maturity index while paired *t*-test was conducted for carbonate: phosphate ratio. Values were mean ± standard deviation (*n* = 3) and *p* < 0.05 was considered as significant as denoted by different letters.

## Discussion

Outgrowth and enzymatic digestion methods are two commonly used methods to obtain dental pulp stem cells (DPSC). Enzymatic digestion allows the release of all types of cells from the pulp tissue, resulting in cells with different morphologies to exist at passage 0 (Nur Akmal et al., 2014; Yildirim, 2013b). While more homogenous cell morphology can be obtained through the outgrowth method, there is the possibility of spontaneous differentiation taking place as the cells that migrate out might grow densely around the tissue. Stem cells are able to spontaneously differentiate and expresses mature cell markers when maintained in confluent state (Dudakovic et al., 2014). As these cells may consist of both mature and stem cells, passaging of cells makes it possible for highly proliferative cells, and cells that can continuously divide, to be obtained (Nur Akmal et al., 2014; Yildirim, 2013b). The fibroblastic morphology obtained is a common dental pulp stem cell morphology as previously reported (Yildirim, 2013b). Although DPSC-OG and DPSC-ED share the same cell morphology, based on the doubling time, we hypothesized that DPSC isolated by means of enzymatic digestion may have more population of highly self-renewing cells. Further analyses were conducted in order to determine the differentiation potential of the cells.

There are several approaches that can be taken in order to differentiate stem cells into chondrocytes which are through 2D monolayer or 3D culture. Chondrocytes cultured as monolayer have the tendency to undergo de-differentiation, show decrement in the expression of collagen II and chondroitin-4-sulphate (Caron et al., 2012; Schnabel et al., 2002), and have higher probability to differentiate and form hypertrophic chondrocytes (Caron et al., 2012). Chondrocyte differentiation conducted in high density culture also has been shown to secrete higher amounts of collagenous matrix (Ronziere et al., 2003). Thus, this study used 3D (micromass) culture for chondrogenic differentiation. sGAG assay was used to further confirm the result of toluidine blue staining. sGAG, which includes keratin sulphate and chondroitin sulphate, is one of the structures that make up proteoglycans. This assay showed that DPSC-ED was able to secrete higher amounts of matrix within a shorter period compared to DPSC-OG.

Osteoblastic differentiation was conducted using proliferation medium supplemented with ascorbic acid and *β*-glycerophosphate. Dexamethasone, a type synthetic glucocorticoid normally added to medium to induce cell differentiation to osteoblast, was not used in this study. While the use of dexamethasone can lead to higher ALP activity and faster rate of mineralization (Coelho & Fernandes, 2000), long term culture of cells in medium containing dexamethasone has been shown to cause cell toxicity and lysis (Kaveh et al., 2011). Ascorbic acid is involved in collagen hydroxylation for formation of a stable collagen (Hitomi & Tsukagoshi, 2013) and *β*-glycerophosphate serves as a source of organic phosphate for mineralized matrix formation (Coelho & Fernandes, 2000). During differentiation to osteoblast in this study, the difference between the proliferative abilities of DPSC-OG and DPSC-ED may have been because the cells were in different stages of differentiation. This was because the stage of osteoblast differentiation influenced the ability of the cells to proliferate (Dallas & Bonewald, 2010; Neve, Corrado & Cantatore, 2011). Cell proliferation would only cease as the osteoblast matured and became embedded inside the matrix (Franz-Odendaal, Hall & Witten, 2006). Von Kossa has been commonly used to analyze osteoblastic differentiation through the detection calcified nodules. However, von Kossa has been shown to give false positive results when the cells were cultured in differentiation medium containing ≥2 mM *β*-glycerophosphate by producing dystrophic mineralization or non-apatitic mineralization (Langenbach & Handschel, 2013). As 10 mM of *β*-glycerophosphate was used in this study, a further analysis using FTIR was utilized to confirm the presence and quality of the Ca/P minerals (Bonewald et al., 2003). These minerals are also known as hydroxyapatite with the formula Ca_10_(PO_4_)_6_(OH)_2_. A lot of information can be derived through analysis using FTIR, which includes, but not limited to the degree of mineralization, presence of carbonated apatites and apatite maturity. Higher degrees of mineralization of cells differentiated *in vitro* could be due to the lack of acid phosphatase activity in the *in vitro* culture. A study on tartrate resistant acid phosphatase knockout mice has shown that there were increases in mineralization leading to osteopetrosis (Hayman & Cox, 2003). Through FTIR analysis, presence of carbonate apatite can also be detected. The incorporation of carbonate in the apatite reduces its crystallinity and increases its solubility due to the weaker bond between Ca–CO_3_, thus helping in the bone turnover in a biological system (Figueiredo, Gamelas & Martins, 2012; Wopenka & Pasteris, 2005). Based on the overall result for osteoblastic differentiation, it was concluded that DPSC-ED showed the best osteoblastic differentiation potential with a faster formation of calcified nodules as shown by von Kossa. Moreover, differentiated DPSC-ED also showed almost the same structural characteristics of bone matrix as calvaria as opposed to differentiated DPSC-OG.

These findings were important as various isolation methods have been used to obtain dental pulp stem cells. Other than the fact that this made comparisons between other studies difficult, the characteristics and differentiation potential of the cells were clearly affected. Besides that, most studies have only used staining to confirm mineralization of osteoblasts. We showed that while the cells can secrete mineralized matrices, the composition was clearly different as opposed to that of bone.

## Conclusion

Dental pulp stem cells isolated through enzymatic digestion (DPSC-ED) and outgrowth (DPSC-OG) showed the cells were *Cd13*^+^, *Cd29*^+^, *Cd105*^+^, *Cd146*^+^, *Cd166*^+^, *Cd34*^−^ and *Cd150*^−^. Compared between these two cells, DPSC-ED showed the best overall performance in terms of *in vitro* expansion and differentiation process to chondrocyte and osteoblast. Chondrocyte differentiation revealed that chondrocyte from DPSC-ED secreted a higher amount of proteoglycan matrix. DPSC-ED were also able to form mineralized, calcified matrix faster than DPSC-OG when differentiated to osteoblast and had better bone matrix formation that resembled calvaria as shown by FTIR. However, *in vitro* and *in vivo* studies involving 3D constructs such as scaffolds were essential to determine the potential of the cells in bone regeneration therapies.

##  Supplemental Information

10.7717/peerj.3180/supp-1 Data S1 Raw data for Fig. 3

10.7717/peerj.3180/supp-2 Data S2 Raw data for Fig. 4 (DPSC-ED)

10.7717/peerj.3180/supp-3 Data S3 Raw data for Fig. 4 (DPSC-OG)

10.7717/peerj.3180/supp-4 Data S4 Raw data for Fig. 5

10.7717/peerj.3180/supp-5 Data S5 Raw data for Fig. 7

## Additional Information and Declarations

### Data Availability

The following information was supplied regarding data availability: The raw data has been supplied as a Supplementary File.
